# Higher levels of nonylphenol were found in human urine and drinking water from rural areas as compared to metropolitan regions of Wuhan, China

**DOI:** 10.1007/s11356-022-20513-6

**Published:** 2022-05-05

**Authors:** Chunyan Xu, Haibo Ling, Chuangang Fan, Luojing Xiang, Shu Zhang, Weiwei Li, Chuan Yi

**Affiliations:** 1 Hubei Academy of Environmental Sciences, Wuhan, Hubei People’s Republic of China; 2 Hubei Key Laboratory of Pollution Damage Assessment and Environmental Health Risk Prevention and Control, Wuhan, Hubei People’s Republic of China; 3 Hubei Provincial Center for Disease Control and Prevention, Wuhan, Hubei People’s Republic of China

**Keywords:** Nonylphenol (NP), Water, Urine, Rural, Urban

## Abstract

The suspected endocrine disruptor nonylphenol (NP) is closely associated with anthropogenic activities; therefore, studies on this compound have been clustered in urban areas. This study investigated the NP concentrations in drinking water sources (*n* = 8), terminal tap water (*n* = 36), and human urine samples (*n* = 127) collected from urban and rural areas in Wuhan, China. The mean concentrations of NP measured in drinking water sources in urban and rural areas were 92.3 ± 7.5 and 11.0 ± 0.8 ng/L (mean ± SD), respectively, whereas the mean levels in urban and rural tap waters were 5.0 ± 0.7 and 44.2 ± 2.6 ng/L (mean ± SD), respectively. Nevertheless, NP was detected in 74.1% and 75.4% of the human urine samples from urban and rural participants, with geometric mean concentrations of 0.19 ng/mL (0.26 µg/g creat) and 0.27 ng/mL (0.46 µg/g creat), respectively. Although the NP concentrations measured in the drinking water sources of urban areas were significantly higher than those in rural areas (*P* < 0.05), the tap water and urine NP concentrations measured in urban areas were unexpectedly lower than those of rural areas (*P* < 0.05). Additionally, this investigation showed that the materials comprising household water supply pipelines and drinking water treatment processes in the two areas were also different. Our results indicated that the levels of exposure to NP in drinking water and human urine in rural areas were not necessarily lower than those in urban areas. Thus, particular attention should be paid to rural areas in future studies of NP.

## Introduction

In recent years, the environmental endocrine disruptor nonylphenol (NP) has been a growing concern for the environmental and scientific community because of its weak estrogenic activities (Jie et al. 2013, 2017). NP, a major degradation product of alkylphenol ethoxylates (Ruczyńska et al. 2020), is widely used in industrial production and is present in many daily necessities, such as detergents and plastics (Jie et al. 2016; Wang et al. 2019). Although some countries and regions, such as the United States of America (USA) and Europe (Pirard et al. 2012), have taken measures to limit the use of NP, in many countries of Asia, NP is still widely produced and used. According to the 2011 annual report of the China Petroleum and Chemical Industry Federation (CPCIF), the production of NP reached as high as 31,434 tons, accounting for 10% of the world’s total production (Jie et al. 2017), which has led to the environment being under great pressure from NP pollution.

As estrogen–mimetic compounds, the main mechanisms by which NP causes damage are by interacting with estrogen receptors and disrupting normal signaling pathways (Laws et al. 2000). Experiments in fish (Shirdel et al. 2020), rats (Kim et al. 2020), *Caenorhabditis elegans* (Cao et al. 2020), and other animals have confirmed the involvement of NP in the disruption of the reproductive system. A population survey also indicated that NP may induce male infertility by exerting a negative impact on sperm quality (Noorimotlagh et al. 2017). Furthermore, many studies have also evaluated the effects of NP on exposed individuals’ offspring, pregnancy, and lactation, which are key periods for the development of the nervous system (Qiu et al. 2019). Therefore, environments and populations exposed to NP have been an area of continuing research interest.

Numerous studies have shown an association between NP concentrations in environmental and anthropogenic activities (Berge et al. 2012; Sharma et al. 2009) demonstrating that NP preferentially enters the environment through urban sources. Thus, rural areas are thought to be of less concern. However, the detection of NP in various environmental media, such as water (Bhandari et al. 2021), soil (Hu et al. 2021), sediment (Careghini et al. 2015), and even in air (Bodziach et al. 2021; Xie et al. 2006), reveals the wide diffusion pathways of NP in the environment. Such a wide diffusion pathway may lead a large proportion of the population to the risk of exposure, which indicates that rural areas are likely to be exposed to this compound as well. However, studies of NP have clustered primarily on urban areas, and NP exposure levels in rural areas are rarely considered.

NP that enters the environment will eventually be incorporated into water matrices in various ways (Bhandari et al. 2021), leading to its widespread presence in aquatic environments, including drinking water sources. Drinking water is considered to be one of the main routes for human exposure (Guenther et al. 2002), as water exposure was demonstrated to be ubiquitous but persistent at low doses (Pirard et al. 2012; Yu et al. 2020). Therefore investigating drinking water is particularly important for evaluating the exposure levels of an area. While mounting research has suggested that NP contamination in environmental water occurs globally, there is still little information about the distribution of NP in drinking water.

Human biomonitoring surveys of compounds are important for determining the exposure level of a population and describing geographical differences, and urine is considered to be an appropriate matrix for NP biomonitoring (Dekant and Volkel 2008). Several countries, such as the USA (Calafat et al. 2005), Belgium (Pirard et al. 2012), Japan (Inoue et al. 2003), Korea (Park and Kim 2017), and China (Li et al. 2013; Peng et al. 2016), conducted surveys on the levels of NP in urine. Those studies showed that urinary NP levels vary significantly according to the population studied, suggesting that the differences in exposure levels may depend on geographical location. In addition, studies of urinary NP levels in different areas, especially between urban and rural areas, are still relatively scarce. Therefore, this study focused on the evaluation of NP exposure in environments and humans between geographical residence areas with distinctly different levels of economic development by examining NP levels in drinking water and human urine in urban and rural areas. The results of this study may provide helpful implications for pollution control and NP health risk management.

## Materials and methods

### Reagents and materials

Standard NP (CAS 25154–52-3, 99.9% purity) and the internal standard 4–n–nonylphenol (4–n–NP) (CAS 104–40-5, > 99% purity) were purchased from Dr. Ehrenstorfer GmbH (Augsburg, Germany). HPLC grade acetone, acetonitrile, and ethyl acetate were obtained from Fisher Scientific (Fair Lawn, NJ, USA). β–Glucuronidase and sulfatase were acquired from Sigma–Aldrich (St. Louis, USA). Analytical grade ammonia, ammonium fluoride, acetic acid, sodium acetate hydrochloric acid, and other auxiliary reagents were all obtained from the National Pharmaceutical Group Chemical Reagent Co. Ltd. (Shanghai, China). Sample extraction and purification were completed with an Oasis HLB cartridge (Milford, MA, USA). In addition, ultrapure water was used throughout the study.

### Preparation of standard solutions

NP and 4–n–NP (1 mg/mL) standard stock solutions were prepared in methanol, and then the stock standard solutions were diluted with methanol (MeOH) to obtain intermediate standard solutions of NP and 4–n–NP (10 μg/mL), which were further diluted and mixed using methanol to prepare 100 ng/mL mixed intermediate standard solutions. A series of mixed standard working solutions were prepared by diluting the mixed intermediate standard solutions with MeOH before use. All solutions described above were stored at 4 °C.

### Study site and population

**Fig. 1:**
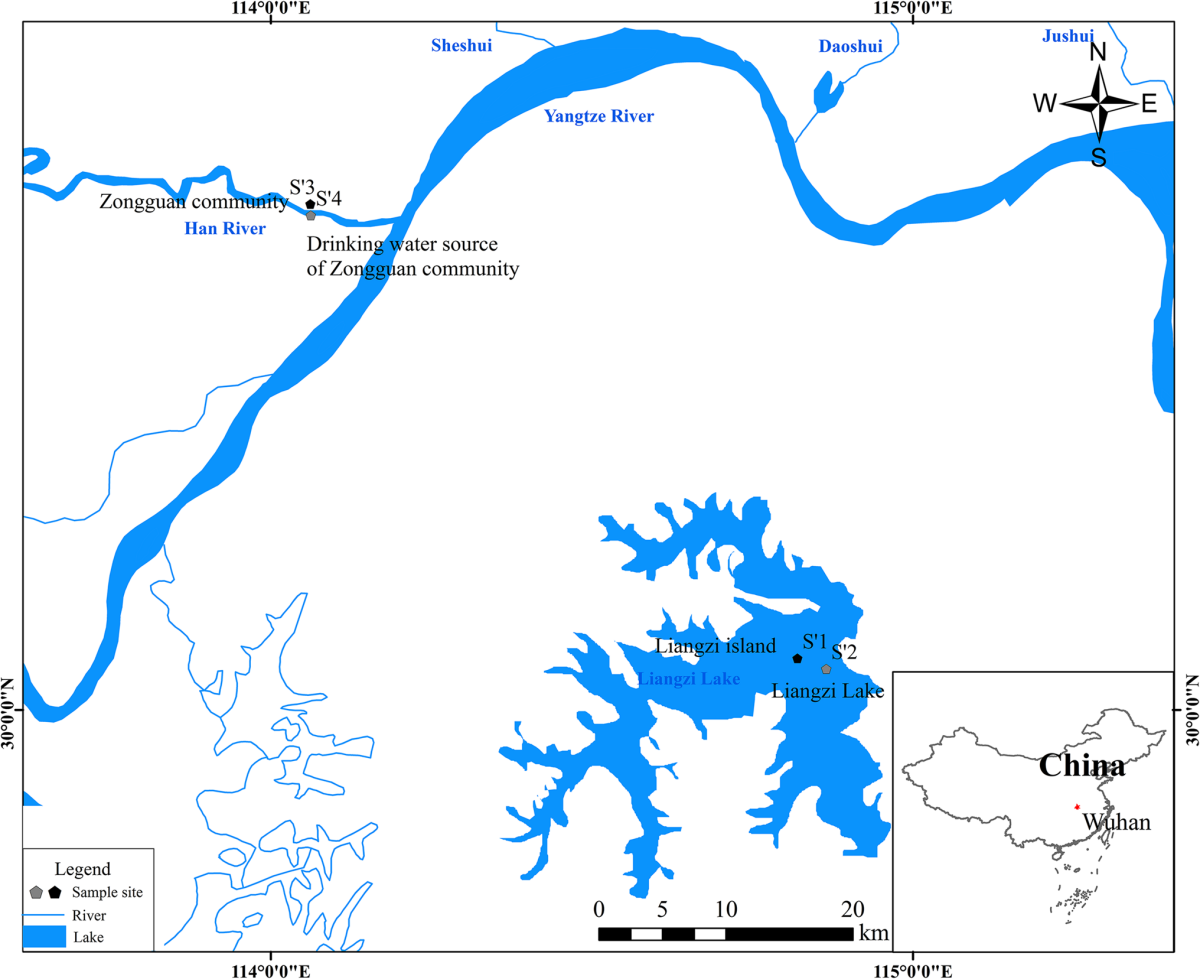
Distribution of sampling locations within the Wuhan metropolitan region, China. S’1 is Liangzi Island, which remains a rural area and tourist destination. S’2 represents the rural drinking water source, Liangzi Lake, a large shallow lake on the outskirts of Wuhan. S’3 represents the Zongguan community, which is located in the middle of the city. S’4 is the urban drinking water source located in the Han River, the largest tributary to the Yangtze River

Two communities were chosen as study sites. These communities were representative of an urban and a rural area in Wuhan, China. One (the Zongguan community, S’3) located in the metropolitan downtown center of Wuhan with well-developed industry and commerce in the surrounding areas, industrial, and domestic sources are all the sources of NP in there. And another (the Liangzi Island, S’1) located on the periphery of Wuhan is an isolated island surrounded by Liangzi Lake without any industrial activities; the permanent residents are only 800, and no industrial source of NP exist in there. The corresponding drinking water sources of these two community were Han River water (S’4)and Liangzi lake water (S’2), respectively. The specific sites of this study are shown in Fig. 1.

Besides, to further study the possible differences between the two areas, an investigation on the materials of the household water supply pipelines and drinking water treatment processes in two areas was further conducted. As shown in Table 1, the water supply pipelines in urban areas were made of stainless-steel pipes, while in rural areas, hard polyvinylchloride (PVC) pipes were used. The urban drinking water treatment process can be briefly described as coagulation-sedimentation-filtration-disinfection, while in rural areas, it was a sedimentation-filtration process.

**Table 1:** Information about material of household water supply pipelines and drinking water treatment process in two areas

Areas	Material of household water supply pipelines	Drinking water treatment process
Liangzi island	Hard polyvinylchloride (PVC)	Sedimentation–Filtration
Zongguan community	Stainless-steel	Sedimentation–Filtration–Coagulation–Sedimentation

A total of 127 participants aged between 18 and 90 years were selected: 58 from the Zongguan communities and 69 from the Liangzi Island. The age range (18–88) was select based on the age distribution of the population in the two areas, besides 3–17 year olds spend most of their day in school where basically not located in study area. Participants were chosen based on the inclusion that they had lived in the area for more than one year and no household water purification facilities had been installed in their home. Informed consent and a brief questionnaire (including data on age, sex, body size, and weight to obtain BMI status) were obtained from all participants before sampling.

### Sample collection

Water samples were taken from 11 locations within the study area. In Zongguan community, 1 location was in drinking water source, and 6 were household taps within participant’s home; in Liangzi island, the specific number were 1 and 3, respectively. Water samples from the drinking water source of the urban and rural areas were collected 100 m upstream from the drinking water intake. The sampling locations of household tap waters were determined as follows. Because the residential building of Zongguan Community was a seven-floor building, which involved a secondary water supply, we randomly selected three household from the first to third floors and three household from the 4th to 7th floors for sampling. The Liangzi Island residential building was a low-level house, and three house was randomly selected for sampling. Of course, all households were selected from participants who had provided urine samples. Two liters of water sample was collected in a 2.5 L brown glass bottle from each location and stored at 4 °C until pretreatment. This process was repeated four times at an interval of 6 h each time, and a total of 44 water samplings were collected.

Urine samples in the two areas was completed on the same day in July 2019 with water sample. All participants were asked to provide first-morning urine voids in a 100 mL polypropylene container. And urine samples were stored at – 80 °C before analysis. The study was reviewed and approved by the Ethics Committee of Hubei Center for Disease Control and Prevention (Wuhan, China).

### Sample preparation

The water samples were prepared in two steps as follows. Filtration: water samples (500–1000 mL) were weighed accurately and passed through a 0.45 μm glass fiber microporous filter membrane to remove suspended solids. Extraction: enrichment was performed on an OASIS HLB column (200 mg, 6 mL) previously conditioned with 5 mL of methanol and 5 mL of water. The analytes were eluted from the SPE cartridges with 10 mL of methanol, which were reconstituted in methanol to a final volume of 0.5 mL and passed through a 0.22 μm polytetrafluoroethylene (PTFE) syringe filter before ultra-performance liquid chromatography tandem mass spectrometry (UPLC–MS/MS) analysis.

The urine samples were prepared briefly as follows: 50 ng of 4–n–NP was added to 2 mL of urine, followed by buffering with 100 µL of sodium acetate (pH 5.5). After the addition of 10 µL of β‐glucuronidase/sulfatase, the mixture was incubated at 37 °C for 3 h. After cooling to room temperature, the dispersed sample solution was centrifuged at 3000 rpm. The supernatant was acidified with hydrochloric acid to a pH of 2–3 and then transferred to OASIS HLB cartridges (60 mg, 3 mL), which were previously conditioned with 5 mL of dichloromethane–methanol (9:1 v/v), 3 mL of methanol, and 3 mL of water (pH 3.0–3.5, adjusted with HCl). Initially, 3 mL of 5% methanol was used for cartridge washing, and subsequently, 5 mL of dichloromethane-methanol (9:1 v/v) was used for elution. Finally, the eluate was evaporated to dryness under a stream of nitrogen and reconstituted in 0.5 mL of methanol for UPLC analysis.

### UPLC–MS/MS conditions

UPLC analyses on water samples were carried out using an Agilent 1290 liquid chromatograph (Agilent, CA, USA) with an Agilent Eclipse Plus C18 column (50 mm*2.1 mm, 1.8 μm). Mobile phases A and B were 1 mmol/L ammonium fluoride aqueous solution and 100% methanol, respectively. The system was run with a gradient program: 85% A held for 0.0–1.0 min, 85% A linear reduction to 5% A from 1.0 to 5.0 min, 5% A held from 5.0 to 8.0 min, and 5% A linear increase to 85% A from 8.0 to 8.1 min. The flow rate was 0.3 mL/min, the column temperature was 35 °C, and the injection volume was 5 µL. Mass spectrometry was carried out on a Triple Quad™ 3500 mass spectrometer (AB SCIEX, MA, USA). The selected parameters were as follows: ion source temperature: 550 °C, spray voltage: 5500 V, curtain gas: 35 psi, impact gas: 7 psi, and atomizing gas and auxiliary heating gas: 55 psi.

UPLC analyses on urine samples were carried out using a Waters Alliance Acquity-E2695 high-performance liquid chromatography system (Waters, MA, USA), and the analytical column was a ZORBAX 300SB–C18 (4.6 mm*150 mm, 5 μm). Mobile phases A and B were methanol and water, respectively. The gradient elution program used was as follows: 35% A–90% A (2 min), 90% A–100% A (3 min) and held for 2 min, and 100% A–35% A (0.5 min) and held for 2.5 min. The flow rate was 0.3 mL/min, the column temperature was 20 °C, and the injection volume was 10 µl. Mass spectrometry was carried out with a Waters Xevo TQD Triple quadrupole mass spectrometer (Waters, MA, USA) using electrospray ionization (ESI) in MRM scan mode. The selected parameters were as follows: capillary voltage: 3.0 kV, desolvation temperature: 350 °C, and desolvation gas: 800 L/h.

The optimum cone voltage, collision energy, and the characteristic ions for analytes and the internal standards are presented in Table 2.

**Table 2:** Selected MRM transitions and optimized potentials of the target compounds

Sample	Analytes	Precursor (m/z)	Product (m/z)	Cone (V)	CE (eV)
Water	NP	219	133	110	45
	4-n-NP		106		
Urine	NP	219	133	50	20
	4-n-NP		106		

### Urinary creatinine adjustment

To normalize individual variation due to the differing hydration states of each participant at the time of sampling (Wang et al. 2016), NP concentrations were adjusted by creatinine levels. Therefore, urinary NP concentrations were presented in the following two forms: (a) as an uncorrected concentration (nanograms per liter, ng/L) and (b) as a concentration corrected according to creatinine levels (micrograms per gram creatinine, µg/g creat).

### Validation study

The analytical method was validated to illustrate the limit of quantitation (LOQ), accuracy, precision, and recovery of the measurements. Standard solutions of NP and 4–n–NP at low, medium, and high concentrations were added to the samples, and 6 parallel samples were set at each concentration to calculate the spiked recovery rate and relative standard deviation (RSD). The limits of detection (LODs) and LOQs of the method were determined by three times and ten times the signal–to–noise ratios, respectively. As shown in Table 3, the precision and accuracy of this method met the requirements of detection.

**Table 3:** Linearity, limit of detection (LOD), method limit of quantitation (LOQ), and the method recoveries and precision for target compounds

Sample	Analytes	Linear range (ng/ml)	*R*^2^	LOD (ng/ml)	LOQ (ng/ml)	Spiked concentrations (ng/ml)	Recovery (%)	RSD (%)
Water	NP	0.1–100	> 0.999	0.002	0.006	4, 10	65.3–105	1.3–1.4
	4-n-NP						71.9–87.8	1.9–2.0
Urine	NP	0.1–100	> 0.999	0.03	0.1	1, 5, 10	96.8–101.2	6.58–8.8
	4-n-NP						95.6–102.3	8.29–9.2

### Statistical analysis

Concentrations below the LOD were counted as half the LOD. All statistical analyses were performed with SPSS software, version 16.0 for Windows (SPSS Inc., USA). An independent-samples *t* test was employed to test the difference in NP concentration in drinking water between the two areas. A nonparametric Mann–Whitney *U* test was used to analyze the differences in urine NP concentration between two areas. Analysis of variance (one-way ANOVA) was used to compare the baseline characteristics of the participants in two areas. Statistical significance was accepted at *P* < 0.05 for all comparisons.

## Results

### NP in drinking water sources and tap waters

**Fig. 2:**
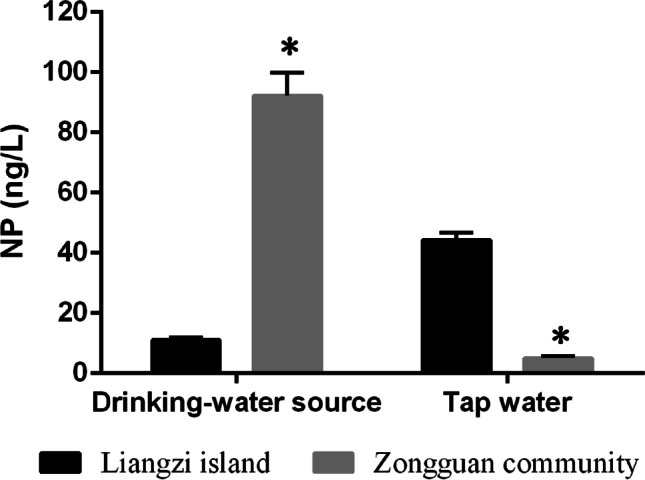
Comparison of the NP concentration among the drinking water source and terminal tap water of the two areas (ng/L). Quantitative analyses of the NP concentrations between the drinking water source and terminal tap water of the two areas. Each bar denotes the mean ± SD. *P < 0.05 as compared to Liangzi Island

The concentration of NP in different water samples across the study area are shown in Table 4. NP was detectable in all 44 water samples with an LOD of 2.0 ng/L. The mean (SD) concentration of NP measured in drinking water sources in urban and rural areas were 92.3 ng/L (7.5) and 11.0 ng/L (0.8), with a range of 82.1–98.9 ng/L and 10.0–11.9 ng/L, respectively. While, in terminal tap water, the average concentration in urban and rural areas were 5.0 ng/L (0.7) and 44.2 ng/L (2.6), with a range of 4.0–6.0 ng/L and 41.7–47.8 ng/L, respectively. In urban areas, the NP concentration in drinking water sources was markedly higher than that of terminal tap water (*P* < 0.01), while in rural areas, the NP concentration in drinking water sources was unexpectedly lower than that of terminal tap water (*P* < 0.01). A comparison of the NP concentrations in the tap water and drinking water sources between the two areas is presented in Fig. 2. The concentration of NP in terminal tap water in urban areas was statistical lower (*P* < 0.05) than that in rural areas, although the NP concentration of drinking water sources in urban areas was significantly higher (*P* < 0.05) than that in rural areas.

**Table 4:** Comparison of NP concentration in drinking water samples in two areas

Sample site	*n*	Range of detected levels of water NP (ng/L)	Water NP concentration (ng/L) Mean (SD)	*P* value
*Liangzi island*				< 0.01
Drinking water source	4	10.0–11.9	11.0 ± 0.8	
Tap water	12	41.7– 47.8	44.2 ± 2.6	
*Zongguan community*				< 0.01
Drinking water source	4	82.1–98.9	92.3 ± 7.5	
Tap water	24	4.0–6.0	5.0 ± 0.7

### Urine NP levels in urban and rural inhabitants

A total of 127 urine samples (*n* = 58 and 69 for the urban and rural areas, respectively) from urban and rural areas of Wuhan, China, of nonoccupationally exposed mixed populations were collected. All participants were categorized in three age groups (≤ 40, 40–60, ≥ 60). Participants were 67.2% and 66.7% female for urban and rural areas, respectively, with the mean (SD) ages of 54.1 (13.5) and 54.1 (10.6) years. The baseline characteristics of the participants in these two areas are presented in Table 5. There were no significant baseline differences between the urban and rural participants in terms of sex distribution, age range, or BMI. The frequency of detection, geometric mean (GM), percentile, and range concentrations for urine NP in the two areas are detailed in Table 6. The detection rates of NP in urban and rural areas were 74.1% and 75.4%, respectively, with an LOD of 0.03 ng/mL. This result suggested that NP exposure to the Wuhan population was quite prevalent. The GM concentration for NP in urban areas was determined to be 0.19 ng/mL (0.26 µg/g creat), ranging from ND (not detected) to 1.69 ng/mL (from ND to 13.59 µg/g creat), while the concentration in rural areas was 0.27 ng/mL (0.46 µg/g creat), with a range of ND to 4.24 ng/mL (from ND to 20.32 µg/g creat). Significant differences (*P* = 0.01) were found between the two geographical areas in terms of urine NP levels. Participants from rural areas exhibited higher urinary NP concentrations than their urban counterparts.

**Table 5:** Baseline characteristics of participants in two areas

	Zongguan community	Liangzi island	*P* value
N	58	69	
Age, n (%)			0.49
≤ 40	8 (13.8)	8 (11.6)	
40–60	31 (53.4)	44 (63.8)	
≥ 60	19 (32.8)	17 (24.6)	
Gender, *n*= (%)			0.95
Female	39 (67.2)	46 (66.7)	
Male	19 (32.8)	23 (33.3)	
Height, mean (SD), cm	160.9 (7.2)	162.5 (8.3)	0.26
Weight, mean (SD), kg	62.4 (13.0)	61.4 (10.3)	0.63
Body mass index, mean (SD)	23.6 (5.3)	23.2 (3.0)	0.58

**Table 6:** Number of individuals (N), frequency of detection, geometric mean, range levels, and percentile for urinary NP in two areas

	Zongguan community	Liangzi island
*N* (Positive Samples %)	58 (74.1%)	69 (75.4%)
Geometric mean μg/ml (μg/g creat)	0.19 (0.26)	0.27 (0.46)
Range μg/ml (μg/g creat)	0.02–1.69 (0.02–13.59)	0.02–4.24 (0.02–20.32)
10th μg/ml (μg/g creat)	0.02 (0.02)	0.02 (0.02)
50th μg/ml (μg/g creat)	0.21 (0.29)	0.34 (0.48)
90th μg/ml (μg/g creat)	0.79 (1.38)	1.03 (2.40)
Mann–Whitney U	1479	
*P* value	0.01	

## Discussion

To fully assess the difference in NP exposure levels between urban and rural areas, we selected both drinking water NP and urinary NP as external and internal exposure indicators, and the levels of both indicators between the two areas were compared. Comparison of the results of the levels of NP in drinking water between the urban and rural areas were noteworthy. In drinking water sources, the NP concentration measured in urban areas was significantly higher than that of rural areas (*P* < 0.05), while the measured NP concentration in terminal tap water of the urban area was unexpectedly lower than that of rural areas (*P* < 0.05). The literature has shown that pollutants from discharged sewage effluent are the main factor that causes NP pollution in drinking water sources (Jie et al. 2017). With regard to the sampling locations in this study, the urban drinking water source was located in the Han River, which is one of the main tributaries to the Yangtze River, with well-developed industries and densely populated neighborhoods in the surrounding area. The Han River has become a main recipient water body for sewage treatment plants. On the other hand, the rural drinking water source was located in Liangzi Lake, where the wetland nature reserve of Hubei Province, where sewage discharge has been prohibited. The significant differences in NP concentration in drinking water sources between the two areas may be due to the fact that the Han River receives more effluents from sewage treatment plants and domestic households, leading to higher NP contamination than its rural counterpart, Liangzi Lake.

As described above, the materials of household water supply pipelines and drinking water treatment processes between the two areas were also different. Many studies have revealed that NP is usually used as a heat stabilizer for PVC and can be released into drinking water from household facilities (Cheng et al. 2015; Loyo-Rosales et al. 2004). Our results showed that the NP concentration in PVC drinking water pipes was higher than that of stainless steel pipes, which is consistent with previous studies (Cheng et al. 2015), verifying that the water could absorb NP from PVC pipes. Furthermore, the urban drinking water treatment process is more sophisticated and can be briefly described as coagulation-sedimentation-filtration-disinfection, while in rural areas, it is simply a sedimentation-filtration process. It has been previously shown that different water treatment processes exhibit different elimination efficiencies for endocrine disruptors (Chen et al. 2013; Gilca et al. 2020). The differences in the drinking water treatment processes between these two areas may be another factor that caused the higher NP concentration in terminal tap water samples in rural areas.

Literature data concerning the levels of NP in drinking water are scarce in comparison with those of environmental waters. The presence of NP in tap water and drinking water sources in both urban and rural areas implied that there was NP contamination in the drinking water in Wuhan. A comparison between other cities and our survey in Wuhan was conducted for the NP concentrations in tap water and drinking water sources. We found that the concentrations measured in the present study, ranging from 4.0 to 96.2 ng/L, were markedly lower than those in other cities in China, such as Shanghai (Ma et al. 2006), Shenyang (Tang et al. 2005), Chongqing (Shao et al. 2002), Zunyi (Jie et al. 2017), Suzhou (Chen 2013), and Taiwan (Dai et al. 2019), but were higher than those in Europe, including Spain (Valcárcel et al. 2018) and Portugal (Carvalho et al. 2015). As NP mainly originates from industrial discharge (Fürhacker et al. 2000; Soares et al. 2008), the relatively slight pollution from NP in Wuhan compared to other reported cities in China might have benefited from the restrictions on industrial production that started in June 2019 and lasted for 5 months, for the sake of the 7th CISM Military World Games taking place later during that period. It was not surprising that the concentration of NP in drinking water in Wuhan was higher than that in other cities of Europe given that NP has been restricted for several years in those districts as a hazard to human and environmental safety, while in Asia, NP is still being used.

The concentrations of NP in urine differs widely in various countries worldwide. For example, Park and Kim (2017) found urinary NP in 83.2% of 1865 Koreans aged 18–69 years with a GM concentration of 3.70 ng/mL; Li et al. (2013) positively detected urinary NP in 100% of 287 children and students aged 3 to 24 years in Guangzhou, China, and the GM concentration was as high as 17.40 ng/mL; and Pirard et al. (2012) found that NP was not detected in any sample in a general Belgian population. The concentration of NP detected in urban (0.19 ng/mL) and rural (0.27 ng/mL) areas of Wuhan was lower than that found in Guangzhou, China, but was much higher than that found in Belgium. The concentrations of NP obtained from different populations can be influenced by many factors, such as the age range studied, geographical differences, and study design (Peng et al. 2016). In terms of age, it can be reasonably believed that teenagers normally use more plastic products than the older population and therefore could be more frequently exposed to NP (Pirard et al. 2012). The concentration of NP detected in Wuhan, which was lower than that found in Korea and Guangzhou, China, may be related to the fact that the survey population in this study was mainly middle-aged, while in the studies from Guangzhou and Korea, (especially Guangzhou) contained mostly teenagers.

Differences in urinary NP levels related to sex, age, BMI, and urban or rural areas are still controversial. The ages of the participants in this study in the two areas were divided into three categories: ≤ 40, 40–60, and ≥ 60 years old. The sex distribution between the populations in the two areas showed no significant difference, as did the age and BMI distribution. The results of the urine samples collected in this study showed that the concentration of NP in rural areas was higher than that in urban areas, and the difference was found to be statistically significant (*P* = 0.01). Literature data of comparative studies regarding urinary NP in areas of different urbanization are scarce, but there are studies on other endocrine factors. Karalius et al. (2014) found that although the release of BPA into the environment has been associated with more urban, industrialized areas, the levels of BPA detected in the urine from rural areas were comparable to those of more urbanized cities, which is consistent with our finding on NP in this study.

Our study has three strengths. First, we included rural areas in our survey and comprehensively assessed the difference in exposure levels between urban and rural areas, even though research on NP is currently concentrated in urban areas. Second, our study first combined both drinking water NP and urinary NP as external and internal exposure indicators to assess the exposure differences between the two areas. Finally, our research has provided more information on the influence of household water supply pipeline materials and drinking water treatment processes on NP levels in drinking water.

On the other hand, our study also has some limitations. The sample size of our survey was small, and the influencing factor of NP in the human body was complex. For instance, more diet and environmental monitoring in the two areas are required to determine whether there are other, different sources of exposure between rural and urban areas, which may account for urinary NP level differences.

## Conclusion

Even if literature on NP body burden and drinking water contamination levels has increased over the past few years, date on NP levels of urinary and drinking water in different areas, especially with different levels of economic development, are still relatively scarce. This work provides the first description of NP found in human urine and drinking waters in two geographic regions of distinctly different economic levels in Wuhan, China. As already described above, despite their high concentration in drinking water source in more industrialized area, the levels of NP detected in the urine and terminal tap waters from rural areas in Wuhan were unexpectedly higher to those from the urban area. The different materials used for household water supply pipelines and the drinking water treatment processes in the two areas may account for the results described above. Results of this study suggest that even in rural areas of a less industrialization levels, NP is common and that future studies of this compound should include areas less associated with NP exposure and pollution.

## Data Availability

The datasets generated during and/or analyzed during the current study are available from the corresponding author upon reasonable request.

## Abbreviations

NP: Nonylphenol
